# Epithelial‐mesenchymal transition‐converted tumor cells can induce T‐cell apoptosis through upregulation of programmed death ligand 1 expression in esophageal squamous cell carcinoma

**DOI:** 10.1002/cam4.1564

**Published:** 2018-05-31

**Authors:** Aung Kyi Thar Min, Hirokazu Okayama, Motonobu Saito, Mai Ashizawa, Keita Aoto, Takahiro Nakajima, Katsuharu Saito, Suguru Hayase, Wataru Sakamoto, Takeshi Tada, Hiroyuki Hanayama, Zenichirou Saze, Tomoyuki Momma, Shinji Ohki, Yusuke Sato, Satoru Motoyama, Kosaku Mimura, Koji Kono

**Affiliations:** ^1^ Department of Gastrointestinal Tract Surgery Fukushima Medical University Fukushima Japan; ^2^ Department of Thoracic Surgery Akita University Graduate School of Medicine Akita Japan; ^3^ Department of Advanced Cancer Immunotherapy Fukushima Medical University Fukushima Japan; ^4^ Department of Progressive DOHaD Research Fukushima Medical University Fukushima Japan

**Keywords:** epithelial‐mesenchymal transition, esophageal squamous cell carcinoma, glycogen synthase kinase‐3β, immunotherapy, programmed death ligand 1

## Abstract

Esophageal squamous cell carcinoma (ESCC) is an aggressive tumor, and it is urgently needed to develop novel therapeutic strategies including immunotherapy. In this study, we investigated the upregulation of the programmed death ligand 1 (PD‐L1) due to epithelial‐mesenchymal transition (EMT) in ESCC using an in vitro treatment system with the EMT inducer, glycogen synthase kinase (GSK)‐3 inhibitor, and we also analyzed the correlation of EMT and PD‐L1 expression in the clinical tumor samples of both tissue microarray (TMA) samples (n = 177) and whole tissue samples (n = 21). As a result, the inhibition of GSK‐3β induces EMT phenotype with upregulated vimentin and downregulated E‐cadherin as well as increased Snail and Zinc finger E box‐binding homeobox (ZEB)‐1 gene expression. Simultaneously, we showed that EMT‐converted ESCC indicated the upregulation of PD‐L1 at both protein (total and surface) and mRNA levels. Of importance, we showed that EMT‐converted tumor cells have a capability to induce T‐cell apoptosis to a greater extent in comparison to original epithelial type tumor cells. Furthermore, the immunohistochemical stains of ESCC showed that PD‐L1 expression on tumor cells was positively correlated with EMT status in TMA samples (*P* = .0004) and whole tissue samples (*P* = .0029). In conclusion, our in vitro and in vivo study clearly demonstrated that PD‐L1 expression was upregulated in mesenchymal type tumors of ESCC. These findings provide a strong rationale for the clinical use of anti‐PD‐1/anti‐PD‐L1 monoclonal antibodies for advanced ESCC patients.

## 1 INTRODUCTION

Esophageal cancer affects more than 450 000 people, and the incidence is increasing worldwide now.^1^ Globally, esophageal cancer is the sixth most common malignancy in men and the sixth most common cause of death from cancer,^2^ and the majority of the cases in Asian countries are esophageal squamous cell carcinoma (ESCC). The overall survival of the patients with advanced ESCC remains still poor despite the multidisciplinary treatment including surgery, chemotherapy, radiotherapy, and molecular‐targeted drug treatment.^3^, ^4^, ^5^, ^6^, ^7^ Therefore, it is urgently needed to develop novel therapeutic strategies for advanced ESCC.

Immunotherapy has recently become an important part of cancer treatment.^8^, ^9^ The most successful immunotherapy in solid human cancers is the administration of immune checkpoint inhibitors, including antibodies against cytotoxic T lymphocyte‐associated antigen 4 (CTLA4), programmed death 1 (PD‐1), and programmed death ligand 1 (PD‐L1). The PD‐L1 expressed on tumor cells binds to PD‐1 on tumor infiltrating lymphocytes (TILs) in tumor microenvironments, allowing tumor cells to escape from immune attack with TILs and leading TILs to apoptosis.^10^, ^11^, ^12^ As there are several clinical trials of the anti‐PD‐1 monoclonal antibody (mAb) induced significant clinical responses among the several types of the refractory tumors,^13^, ^14^, ^15^, ^16^, ^17^ the inhibition of PD‐1/PD‐L1 interaction by therapeutic mAb would be a new promising strategy for ESCC.

PD‐L1 expression within tumor microenvironment is regulated by the innate immune resistance which includes oncogenic pathways on tumor cells and acquired immune resistance such as IFN‐γ, signaling pathways, and transcriptional factors.^18^, ^19^, ^20^, ^21^, ^22^, ^23^ Furthermore, it has recently been reported that microRNA‐200/Zinc finger E box‐binding homeobox (ZEB)‐1 axis controls both epithelial‐mesenchymal transition (EMT) and PD‐L1 expression in tumor cells.^24^ EMT is the process which allows tumor cells to enter the bloodstreams and lymphatics by changing their phenotype into mobile mesenchymal form to undergo distant metastasis.^25^ The tumor cells undergoing EMT also express PD‐L1, which is one of the essential factors of immune evasion, on the way to the distant metastatic sites.

In this study, therefore, ESCC cell lines treated with glycogen synthase kinase (GSK)‐3 inhibitor were used to determine whether EMT conversion could induce upregulation of PD‐L1 along with increased T‐cell apoptosis. Also, we evaluated the correlation of EMT status with PD‐L1 expression in ESCC samples, using 2 independent cohorts of tissue microarray (TMA) and whole tissues sections obtained from a total of 198 patients with ESCC who underwent surgery.

## 2 MATERIALS AND METHODS

### 2.1 Tumor cell lines

ESCC cell lines, KYSE30 and KYSE110, were purchased from the Japanese Collection of Research Bioresources Cell Bank (Osaka, Japan). SW480 and HCT116, Colon cancer cell lines, were purchased from the American Type Culture Collection (Manassas, VA, USA) and RIKEN Bio Resource Center (Ibaraki, Japan), respectively. All cell lines were cultured in RPMI‐1640 (Sigma‐Aldrich, St. Louis, MO, USA) with 10% fetal bovine serum (Biological Industries Kibbutz Beit Haemek and, Haifa, Israel) and 1% antibiotic‐antimycotic (Thermo Fisher Scientific, Waltham, MA, USA).

### 2.2 EMT induction

EMT induction was performed using GSK‐3 inhibitor, SB‐415286 (S3567_SIGMA; Sigma‐Aldrich) which also inhibits the kinase activity of GSK‐3β,^26^ dissolved with DMSO, and DMSO was used as a vehicle control. The inhibition of the GSK‐3β induces the upregulation of Snail gene which leads tumor cells to EMT and SB‐415286 promotes the Snail gene transcription.^27^, ^28^ Tumor cells were grown to subconfluency and treated with 5 or 50 μmol/L of SB‐415286 for 12‐72 hours. After 48 hours incubation with SB‐415286, tumor cells were morphologically checked by microscope and were analyzed by Western blot and flow cytometry or were used for coculture experiments. For qRT‐PCR, tumor cells were incubated with SB‐415286 for 12‐72 hours.

### 2.3 Western blotting

The cells were collected using simple scraping method, and the pellets were lysed in RIPA buffer (Thermo Fisher Scientific) with phenylmethanesulfonyl fluoride (Santa Cruz Biotechnology, Santa Cruz, CA, USA), protease inhibitor cocktail (Santa Cruz Biotechnology) and sodium orthovanadate (Santa Cruz Biotechnology). The protein concentration of the lysates was measured with Bradford Reagent (Bio‐Rad, Hercules, CA, USA). Electrophoresis was performed using X cell sure lock port with 4%‐20% Tris‐glycerin gel (Invitrogen, Carlsbad, CA, USA), and the protein samples were transferred to Polyvinylidene difluoride (PVDF) membrane using iBlot 2 (Invitrogen). PVDF membranes were blocked with 1% Tris‐buffered saline containing Tween (Cell Signaling Technology, Danvers, MA, USA) with 2% nonfat dry milk. The PVDF membranes were incubated with β‐actin mouse antibody (1:2000; Santa Cruz Biotechnology) in TBST for 1 hour and then incubated with the HRP‐linked anti‐mouse antibody (1:2500; Santa Cruz Biotechnology) in TBST for 1 hour. To detect PD‐L1, the PVDF membranes were incubated with XP(R) Rabbit, PD‐L1 antibody (clone E1L3N; Cell Signaling Technology) at 1:1000 dilution in TBST overnight at 4°C. After incubating with appropriate secondary antibodies, the protein signals were visualized with SuperSignal West Pico Chemiluminescent Substrate (Thermo Fischer) for 5 minutes according to manufacturer’s protocol, and images were captured by LAS‐4000 luminescent image analyzer mini (Fujifilm, Tokyo, Japan).

Detecting how GSK‐3 inhibitor, SB‐415286, downregulates GSK‐3β subunit, the same method of electrophoresis and membrane transfer was carried out with the protein lysate of KYSE 30 or KYSE110 cell line. Then, the membrane was blocked with 5% BSA in TBST overnight at 4°C. The membrane was later incubated with the primary anti‐GSK‐3 (alpha + beta) (phospho Y216 + Y279) antibody (Abcam, Cambridge, UK) for 2 hours in the room temperature. The secondary antibody and the visualization of protein signals were same as above.

To detect proteins of EMT status, tumor cell lines were detached using enzyme‐free dissociation buffer and then lysed with cell lysis cocktail containing cell lysis buffer (Sigma‐Aldrich), protease inhibitor (Sigma‐Aldrich), and phosphatase inhibitor (Sigma‐Aldrich). An equal amount of protein samples was loaded on NuPAGE Novex 4%‐12% Bis‐Tris precast gels (Thermo Fisher Scientific) and transferred to PVDF membranes using the iBlot Dry Blotting System (Thermo Fisher Scientific). PVDF membranes were blocked with either 5% nonfat milk for 2 hours at room temperature and then incubated with primary antibodies overnight at 4°C. PVDF membranes were incubated with anti‐rabbit or anti‐mouse HRP‐linked antibodies (Cell Signaling Technology). Blots were visualized by SignalFire^™^ ECL Reagent (Cell Signaling Technology), and images were captured by ImageQuant LAS500 (GE Healthcare Life Science, Princeton, NJ, USA). The following antibodies were used as primary antibodies: E‐cadherin (1:5000; Santa Cruz Biotechnology), vimentin (1:5000; Santa Cruz Biotechnology), Snail (1:100; Santa Cruz Biotechnology), and Actin (1:5000; Cell Signaling Technology).

### 2.4 Flow cytometry

Following manufacturer’s flow cytometry preparation protocol, in brief, cells were stained with antibodies for 15 minutes at room temperature in the dark. After incubation, stained cells were analyzed using flow cytometry. The following antibodies were used for staining: PE‐conjugated anti‐human CD274 (B7‐H1; PD‐L1) (eBioscience, San Diego, CA, USA), APC‐H7 conjugated anti‐human CD3 (Becton Dickinson Bioscience, San Jose, CA, USA), PE‐conjugated Annexin V (Becton Dickinson Bioscience), PerCP‐Cy5.5 conjugated 7‐Aminoactinomycin D (7‐AAD) (Becton Dickinson Bioscience), and APC‐conjugated E‐cadherin (Biolegend, San Diego, CA, USA). Isotype‐matched immunoglobulin served as a negative control, and staining intensity was measured by FACSCanto II flow cytometer (Becton Dickinson Bioscience), and data were analyzed using Flowjo Software (Flowjo, Ashland, OR, USA).

### 2.5 qRT‐PCR analysis

TRIzol reagent (Invitrogen) was used to collect the specimen, and RNA isolation was performed according to standard protocol. cDNA was synthesized using SuperScript III First‐Strand Synthesis System for RT‐PCR (Invitrogen) on Veriti 96‐well thermal cycler (Applied Biosystems, Foster City, CA, USA). The PCR analysis was performed using TaqMan Gene Expression Assays (Applied Biosystems) with CDH1(E‐cadherin), Snail, CD274 (PD‐L1), and ZEB‐1 according to the protocol from the manufacturer. GAPDH was used as the endogenous control. The plate was analyzed with 7500 Real‐time PCR system (Applied Biosystems). The relative expression levels were normalized to GAPDH and calculated by the 2^−ΔΔCt^ method. The following probes were used for PCR: *GAPDH* (hs99999905_m1), *CDH1(E‐cadherin)* (hs01023894_m1), *SNAI1*(*Snail)* (hs00195591_m1), *CD274(PDL1)* (hs01125301_m1), and *ZEB1* (hs00232783) (all from Applied Biosystems).

### 2.6 Coculture experiment

Peripheral blood mononuclear cells (PBMCs) were isolated from the fresh blood of healthy donor by density gradient centrifugation using Ficoll‐Paque (GE Healthcare, Uppsala, Sweden) The PBMCs were cultured in AIM‐V medium (Thermo Fisher Scientific) with 200 IU/mL of human IL‐2 (Sigma‐Aldrich) for 7 days. Fresh medium and IL‐2 were replenished every 3 days. After 7 days of culture, the IL‐2 activated lymphocytes including T cells, expressing PD‐1 (data not shown),^29^ were used for coculture experiments. The IL‐2 activated lymphocytes were cocultured with the GSK‐3 inhibitor or DMSO‐treated tumor cells at 1:1 ratio in 24‐well plates for 48 hours. After 48‐hour incubation, the proportion of apoptotic CD3‐positive cells, T cells, were analyzed with Annexin V and 7‐AAD using flow cytometry. The 3 independent coculture experiments were performed. This study was also approved by the Institutional Review Board of Fukushima Medical University.

### 2.7 Patient’s samples

One hundred and seventy‐seven TMA samples (3 cores each from 177 tumors) of patients with ESCC, who underwent esophagectomy between January 2000 and December 2011, were provided from Department of Thoracic Surgery, Akita University Graduate School of Medicine.^30^ We also obtained 21 formalin‐fixed paraffin embedded (FFPE) whole tissue samples of ESCC, which were surgically resected at Department of Gastrointestinal Tract Surgery, Fukushima Medical University between April 2003 and January 2016. The study was conducted in accordance with the Declaration of Helsinki and was approved by the ethics committee of the Akita University School of Medicine and Fukushima Medical University School of Medicine.

### 2.8 Immunohistochemistry (IHC)

Both TMA and whole tissue samples of ESCC (4 μm thick) were deparaffinized in xylene and rehydrated through a graded ethanol series. Endogenous peroxidase activity was inactivated by incubation in 0.3% hydrogen peroxide in methanol. For E‐cadherin staining, antigens on the samples were retrieved using autoclaving for 5 minutes in 10 mmol/L citrate buffer solution (105°C, pH 6.0), and the samples were incubated with the primary mouse monoclonal antibody for E‐cadherin (1:200, NCH‐38, Dako) at 4°C overnight and detected a horseradish peroxidase (HRP)‐coupled anti‐mouse polymer (Envision+System‐HRP, Dako, Belgium) followed by incubation with diaminobenzidine (DAB, Dojindo, Maryland). For vimentin staining, the heat‐induced epitope retrieval (HIER) steps were not necessary; therefore, the samples were directly incubated with the primary rabbit polyclonal antibody for vimentin (SP20, 1:400; Nichirei Bioscience, Tokyo, Japan) and detected by a HRP‐coupled anti‐rabbit polymer (Envision+System‐HRP, Dako) followed by incubation with DAB (Dako). For PD‐L1 staining, antigen retrieval was performed using autoclaving for 10 minutes in TE buffer (120°C, pH 9.0) and incubated with the primary rabbit polyclonal antibody for PD‐L1 (E1L3N, 1:400; Cell Signaling). The detection steps for PD‐L1 were same as vimentin.

TMA samples (triplicate cores from 1 tumor) were evaluated with immunoreactivity score (IRS) as follows: Staining intensity score where, weak staining = 1, moderate staining = 2, strong staining = 3, and staining percentage score where <5% of staining area = 0, 5%‐25% of staining = 1, 26%‐50% of staining = 2, 51%‐75% of staining = 3, and >75% of staining = 4. Multiplication of these 2 scores (intensity score and percentage score) resulted in the IRS score which varies from 0 to 12. From IRS score, E‐Cadherin ≥5 is considered as positive, and vimentin ≥4 is considered as positive.^31^ PD‐L1 is considered as positive if ≥5% of area is positive with weak, moderate, and strong staining intensity; therefore, IRS score 0 is considered as negative, and the rest are considered as positive.^32^

Evaluating of FFPE tissue samples was done using H‐score which was calculated by following formula: 1 × (percentage of cells showing weak staining) + 2 × (percentage of cells showing moderate staining) + 3 × (percentage of cells showing strong staining). Definition of EMT phenotype is as low E‐cadherin expression with H‐score ≤200 and high vimentin expression with H‐score ≥30. For PD‐L1, IHC was evaluated from intensity and proportion of membranous staining with or without cytoplasmic staining, and it was scored as 0 = <5% of tumor cells, 1 = weak stain in ≥5% of tumor cells, 2 = moderate stain in ≥5% of tumor cells, 3 = strong stain in ≥5% of tumor cells. Therefore, scores 1, 2, and 3 were considered as PD‐L1 positive.^33^ The evaluation was performed by SK and AT in separately without prior knowledge of clinicopathological data.

### 2.9 Statistics

Statistical differences between 2 groups were evaluated by Fischer’s exact test using GraphPad Prism 6 software (GraphPad Software Incorporation, CA).

The Student *t* test was used to show the significant differences between 2 pair groups or independent groups with the same variables. The standard mean error (SEM) was also calculated and used as the error bar.

## 3 RESULTS

### 3.1 EMT induced by GSK‐3 inhibitor

**Figure 1:**
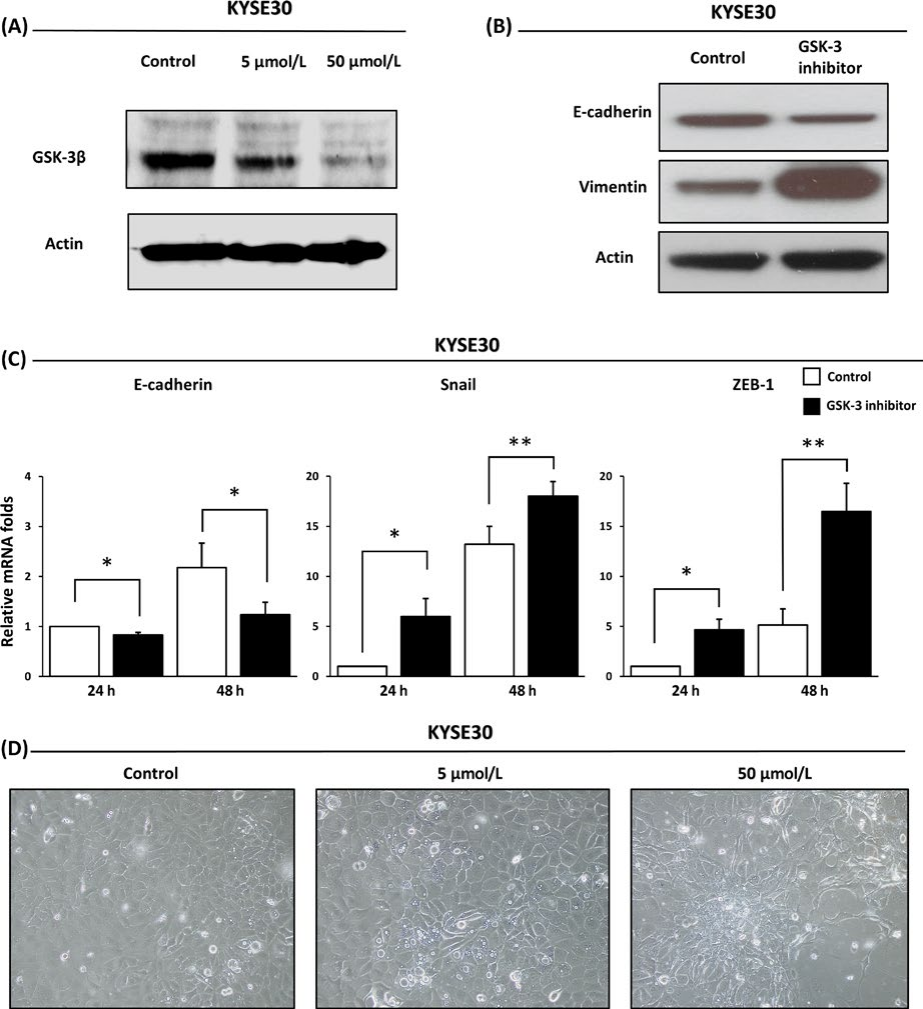
Treatment with GSK‐3 inhibitor induces epithelial‐mesenchymal transition in KYSE30, esophageal squamous cell carcinoma cell line. (A) GSK‐3 inhibitor, SB‐415286, reduces the expression of the GSK‐3β protein in a dose‐dependent manner (Control, 5 μmol/L, 50 μmol/L). (B) Western blot analysis showed the downregulation of E‐cadherin, epithelial marker, and the upregulation of vimentin, mesenchymal marker, after exposure to 50 μmol/L of GSK‐3 inhibitor. (C) QT‐PCR analysis showed the significant downregulation of E‐cadherin gene expression, epithelial marker, and the significant upregulation of Snail as well as ZEB‐1 gene expression, mesenchymal marker, at different time points (24 and 48 h) after exposure to the 50 μmol/L of GSK‐3 inhibitor. DMSO was used for untreated control. Three independent experiments were performed. (D) Microscopic images showed the morphological changes after treatment with a GSK‐3 inhibitor (Control, 5 μmol/L, 50 μmol/L) for 48 h. Spindle‐shaped cells with loss of cell to cell adhesion were seen in 50 μmol/L treatment. *<.05, **<.01

We induced EMT in ESCC cell lines, KYSE30 and KYSE110, and colon cancer cell lines, HCT116 and SW480, using GSK‐3 inhibitor, SB‐415286. As a representative data, the results of KYSE30 were shown in Figure 1. At first, we proved that SB‐415286 reduced the expression of the GSK‐3β protein in a dose‐dependent manner in KYSE30 cell line. (Figure 1A) and KYSE110 cell line (Figure S1a). Tumor cell lines treated with GSK‐3 inhibitor showed the significant downregulation of E‐cadherin and the significant upregulation of mesenchymal genes vimentin and Snail in Western blot and/or PCR analysis (Figure 1B,C and Figure S1b and S2) and the morphological changes including spindle‐like morphology and mesenchymal‐like phenotype with loss of cell to cell adhesion (Figure 1D and Figure S1c), these results showed that GSK‐3 inhibitor was inducing the EMT in these tumor cell lines.

### 3.2 Upregulation of total as well as surface PD‐L1 expression after EMT

**Figure 2:**
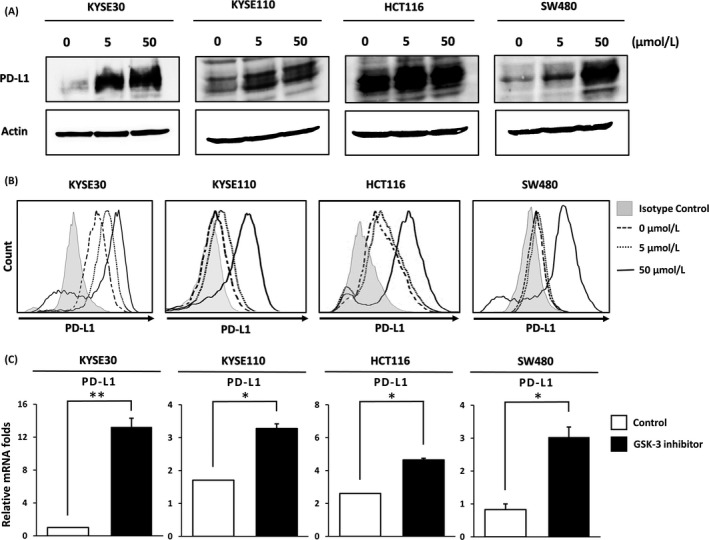
PD‐L1 was upregulated in total protein, surface expression, and gene expression in tumor cell lines treated with the GSK‐3 inhibitor. (A) After exposure to GSK‐3 inhibitor for 48 h, Western blot analysis showed the upregulation of total PD‐L1 protein in a dose‐dependent manner in esophageal squamous cell carcinoma cell lines, KYSE30 and KYSE110, and colon cancer cell lines, HCT116 and SW480. (B) Flow cytometry analysis showed the upregulation of surface expression of PD‐L1 after GSK‐3 inhibitor treatment (Control, 5 μmol/L, 50 μmol/L) for 48 h in tumor cell lines. (C) The significant upregulation of PD‐L1 gene expression in tumor cell lines treated with GSK‐3 inhibitor at 24 h. DMSO was used for untreated control. *<.05, **<.01

After EMT conversion using SB‐415286, we examined the PD‐L1 expression in KYSE30, KYSE110, HCT116, and SW480. Both total amount of PD‐L1 protein and surface expression of PD‐L1 in EMT‐converted tumor cells were increased in a dose‐dependent manner (Figure 2A,B). These results suggested that total amount of PD‐L1 protein as well as the surface expression of PD‐L1 was significantly increased in EMT‐converted tumor cells regardless of tumor type. PCR analysis also showed an increment of both gene expressions of PD‐L1 (Figure 2c, Figure S3) and ZEB‐1 in GSK‐3 inhibitor‐treated group (Figure 1c, Figure S2). To show the relationship between EMT conversion and PD‐L1 expression, we used the double staining of E‐cadherin and PD‐L1 on EMT‐converted tumor cells and the control. The EMT‐converted tumor cells showed the upregulation of PD‐L1 and the downregulation of E‐cadherin at the same time (Figure S5).

### 3.3 EMT‐converted tumor cells induced immune suppression

**Figure 3:**
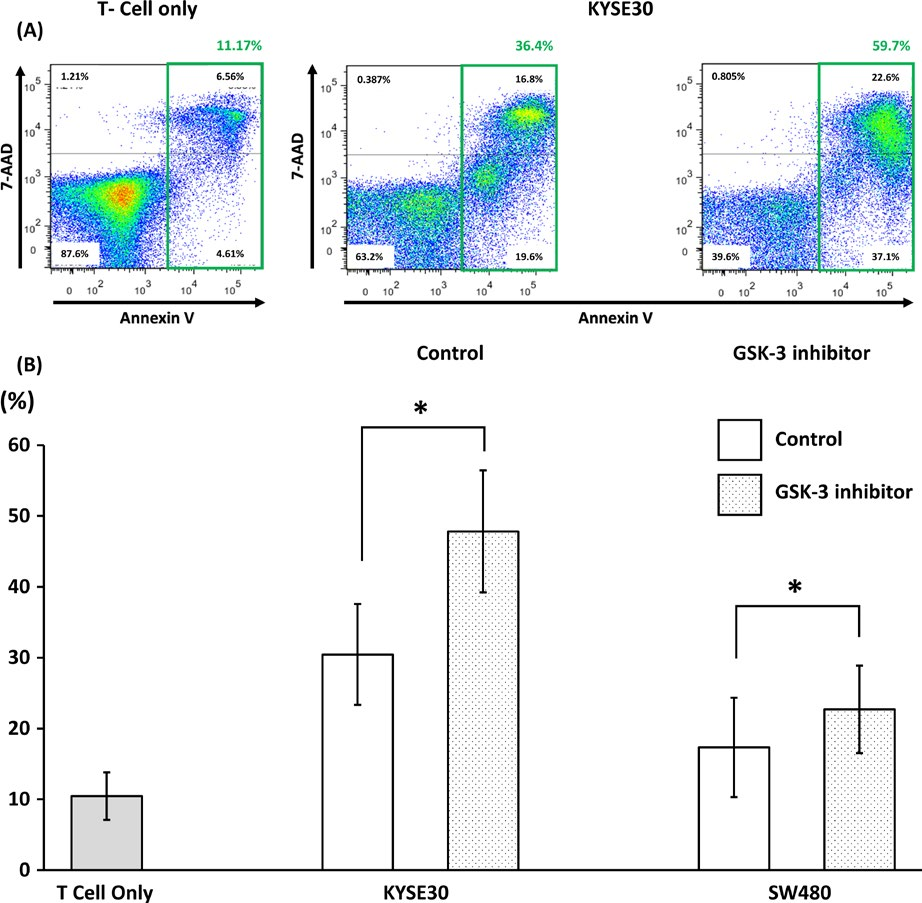
The apoptosis of IL‐2‐activated T cells was induced by the coculture with GSK‐3 inhibitor‐treated tumor cells. (A) The proportion of apoptotic cells was determined using Annexin V and 7‐AAD staining, and the representative figure of 3 independent experiments was shown. The proportion of apoptotic IL‐2‐activated T cells without tumor cells was 11.1%. The proportion of apoptotic IL‐2‐activated T cells was increased in the coculture with GSK‐3 inhibitor‐treated KYSE30 (59.7%), compared to the coculture with untreated KYSE30 (36.4%) DMSO was used for the untreated control. (B) The summery of 3 independent experiments showed the significant difference between control and GSK‐3 inhibitor‐treated groups, of KYSE30 (*P* = .028) and SW480 (*P* = .041) cell lines, respectively. A paired Student’s *t* test was used for the statistical analysis. *<.05

As the EMT conversion using SB‐415286 upregulated the surface expression of PD‐L1 on tumor cells, we next performed coculture experiment using EMT‐converted tumor cells and IL‐2‐activated T cells expressing PD‐1 to examine whether an increment of surface PD‐L1 expression on tumor cells functionally induces T‐cell apoptosis. The proportion of apoptotic T cells after coculture with EMT‐converted tumor cells were analyzed using flow cytometry. To analyze the proportion of apoptotic IL‐2‐activated T cells, CD3‐positive cells were gated out of all cells from culture‐well, and the proportion of Annexin V and 7‐AAD‐positive cells were measured in gated population (Figure 3A and Figure S4). The results from 3 independent experiments showed the significant difference between GSK‐3 inhibitor‐treated and nontreated groups in both KYSE30 cell line and SW480 cell line with *P* = .028 and *P* = .041, respectively (Figure 3B). This result indicated that the surface expression of PD‐L1 on the EMT‐converted cancer cells functionally led T cells expressing PD‐1 to apoptosis.

### 3.4 The PD‐L1 expression on tumor cells was correlated with EMT status in ESCC

**Figure 4:**
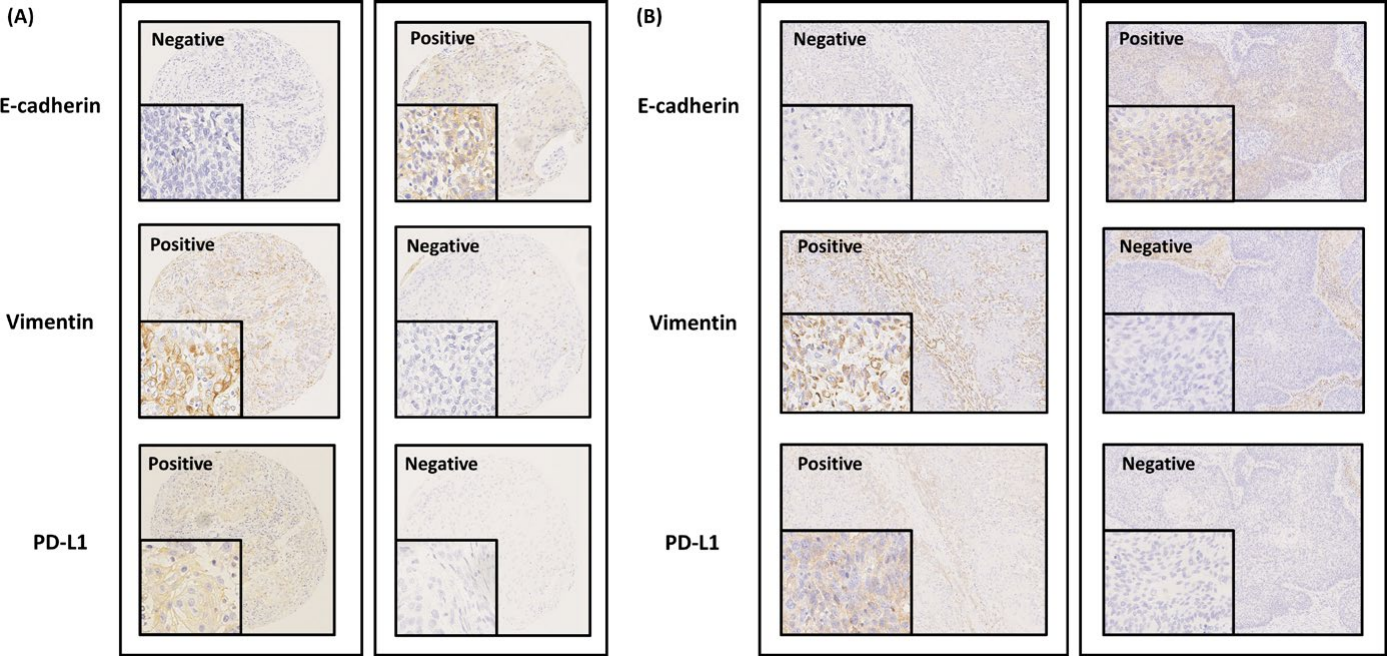
Representative IHC for PD‐L1, E‐cadherin, and vimentin in esophageal squamous cell carcinoma (ESCC). PD‐L1 was expressed in both tissue microarray samples (A) and formalin‐fixed paraffin embedded tissue samples (B) of ESCC. A case with E‐cadherin negative and vimentin positive was considered as epithelial‐mesenchymal transition (EMT) (+), EMT status, which was expressing PD‐L1 ((A) left, (B) left). On the other hand, a case with E‐cadherin positive and vimentin negative was considered as EMT (−), epithelial phenotype, which was not expressing PD‐L1 ((A) right, (B) right). Serial sections in the individual sample were used at a magnification of 10×, together with 40× magnification in small boxes showing the cell staining

**Figure 5:**
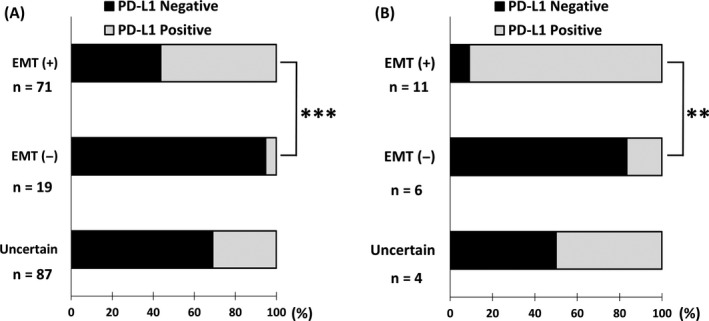
PD‐L1 expression was increased in esophageal squamous cell carcinoma (ESCC) with epithelial‐mesenchymal transition (EMT) status. The significant correlation between EMT status and PD‐L1 expression in ESCC was seen in tissue microarray samples (A) (*P* = .0004) and formalin‐fixed paraffin embedded tissue samples (B) (*P* = .0029). Uncertain cases, which were both E‐cadherin and vimentin positive cases and both negative cases, were excluded from statistical analysis. Fischer’s exact test was used for the evaluation. **<.01, ***<.001

In order to examine the correlation between EMT status and the PD‐L1 expression on tumor cells, IHC for E‐cadherin, vimentin, and PD‐L1 was performed in TMA and FFPE tissue samples of ESCC. Clinicopathologic features of the patients were summarized in the Table 1, and the representative immunohistochemical stains were shown in Figure 4. In both TMA and FFPE tissue samples, the cases with E‐cadherin negative and vimentin positive were considered as EMT (+), while E‐cadherin positive and vimentin negative were considered as EMT (−), and both positive cases and both negative cases were considered as uncertain. As a result, the proportion of PD‐L1‐positive cases on tumor cells was positively correlated with EMT (+) status in TMA samples (*P* = .0004) (Figure 4A and 5A) and in FFPE tissue samples (*P* = .0029) (Figure 4B and 5B). Collectively, the IHC study confirmed the observation that EMT of tumor cells strongly associated with upregulation of PD‐L1 expression in ESCC.

**Table 1:** Patient and tumor characteristics

	Akita MU (n = 177)	Fukushima MU (n = 21)
Age at surgery (years)		
Mean	65	66
Gender		
Male	153	20
Female	24	1
Depth of invasion		
T1a	0	7
T1b	0	8
T2	31	1
T3	137	5
T4a	9	0
Lymph node metastasis		
N0	49	12
N1	55	9
N2	42	0
N3	31	0
Pathological stage^a^		
IA	0	5
IB	10	8
IIA	37	0
IIB	7	2
IIIA	57	4
IIIB	30	0
IIIC	36	0
IV	0	1
Tumor differentiation		
Not poor	120	16
Poor	57	5

a 7th edition of the UICC TNM classification of malignant tumors (ESCC).

## 4 DISCUSSION

The treatment against metastatic ESCC is still challenging, and immunotherapy with anti‐PD‐1 mAb is becoming a promising novel strategy,^3^, ^34^, ^35^, ^36^, ^37^ in which inhibition of PD‐L1 and PD‐1 interaction with the anti‐PD‐1 mAb resulted in the significant clinical response in the early phase clinical trial for advanced ESCC. EMT conversion of tumor cells is known to be one of the major steps for metastasis, and it has been reported that PD‐L1 expression may be partially regulated by EMT‐related gene.^24^, ^33^, ^38^ Therefore, we were interested in investigating the correlation between EMT status and PD‐L1 expression in ESCC. In the present study for ESCC, we showed a significant positive correlation between EMT status and PD‐L1 expression in ESCC cell lines and clinical samples.

It has been reported that the upregulation of PD‐L1 on tumor cells can be mediated by several factors including oncogenic pathway, epigenetic factors, and acquired immune response such as IFN‐γ.^18^, ^19^, ^20^, ^21^, ^22^, ^23^, ^39^ EMT‐inducing transcriptional factors are Snail 1/2, Twist, and ZEB‐1/2, which are controlled by several signaling pathways. Among these signaling pathways of EMT, the most prominent pathway was reported to be TGF‐β signaling pathway, which inhibited GSK‐3β that was located at the end of PI3K‐Akt pathway. The inhibition of GSK‐3β can induce the upregulation of Snail, and subsequently, E‐cadherin expression was downregulated, leading to mesenchymal phenotype.^28^, ^40^, ^41^, ^42^ In the present study, we used SB‐415286 for EMT conversion in ESCC and our results clearly showed that GSK‐3β inhibition induced EMT conversion with upregulation of Snail and ZEB‐1, leading to phenotypic alteration with downregulated E‐cadherin and upregulated vimentin in ESCC cell lines. As a result of EMT conversion, the present study consistently showed the upregulation of PD‐L1 on ESCC cells at both protein (total and surface) and mRNA levels in comparison to untreated original cell lines. Also, other regulatory factors for PD‐L1 expression including cell proliferative pathway, epigenetic factors, and acquired immune response such as IFN‐γ^18^, ^19^, ^20^, ^21^, ^22^, ^23^, ^39^ are needed for the further investigation.

In order to further confirm the above findings, we performed IHC with PD‐L1, vimentin, and E‐cadherin staining using TMA samples and FFPE tissue samples of ESCC. High E‐cadherin score and low vimentin score were considered as epithelial type tumor, while low E‐cadherin score and high vimentin score were considered as a mesenchymal type tumor in the present study. As a result, mesenchymal type tumors significantly expressed high PD‐L1 expression in ESCC tumor samples in comparison with epithelial type. Collectively, our in vitro and in vivo study clearly confirmed that PD‐L1 expression was upregulated in mesenchymal type tumors of ESCC.

As a significant novel finding in the present study, we showed that EMT‐converted tumor cells have a capability to induce T‐cell apoptosis to a greater extent in comparison with original epithelial type tumor cells. This observation implicated that although tumor cells could be primarily targeted by CTLs, EMT‐converted tumor cells with upregulated PD‐L1 expression make CTLs’ apoptosis through interaction between PD‐L1 and PD‐1.^11^, ^12^, ^43^ In this situation, EMT‐converted tumor cells could escape from the immune system including CTLs‐mediated lysis and reach the distant metastatic sites to establish new colonies. Although further study will be needed to resolve the relationship between circulating tumor cells and cytotoxicity of CTLs, treatment with anti‐PD‐1/anti‐PD‐L1 mAb could be a promising strategy for metastatic ESCC.

As described above, inhibition of GSK‐3 also induced upregulation of ZEB‐1, which was an EMT‐inducing transcriptional factor. ZEB‐1 suppressed microRNA‐200 that inhibited PD‐L1 expression in microRNA‐200/ZEB‐1 axis.^24^ In the present study, the upregulation of PD‐L1 and ZEB‐1 gene expression was simultaneously observed in tumor cell lines. According to the previous reports, the p53 was another possible pathway for PD‐L1 regulation through microRNA‐34.^21^, ^44^ Therefore, it is currently believed that there is a complicated situation between EMT conversion induced by GSK‐3 inhibitors and PD‐L1 regulation, and it remains unknown which factors are located as upper‐stream events. In the present study, although we showed the simultaneous downregulation of the epithelial marker E‐cadherin and the upregulation of PD‐L1 on the tumor cells, further studies are required to investigate the causative relationship between EMT conversion and PD‐L1 expression.

In conclusion, our in vitro and in vivo study clearly confirmed that PD‐L1 expression was upregulated in mesenchymal type tumors of ESCC. These findings provide a strong rationale for clinical use of anti‐PD‐1/anti‐PD‐L1 mAb for patients with advanced ESCC.

## CONFLICT OF INTEREST

The authors have no conflict of interest to declare.
