# Supplementary figures and images for: Epithelial‐mesenchymal transition‐converted tumor cells can induce T‐cell apoptosis through upregulation of programmed death ligand 1 expression in esophageal squamous cell carcinoma
Source: Cancer Med. 2018 May 31;7(7):3321–30. doi: 10.1002/cam4.1564 (PMC6051241; doi:10.1002/cam4.1564)

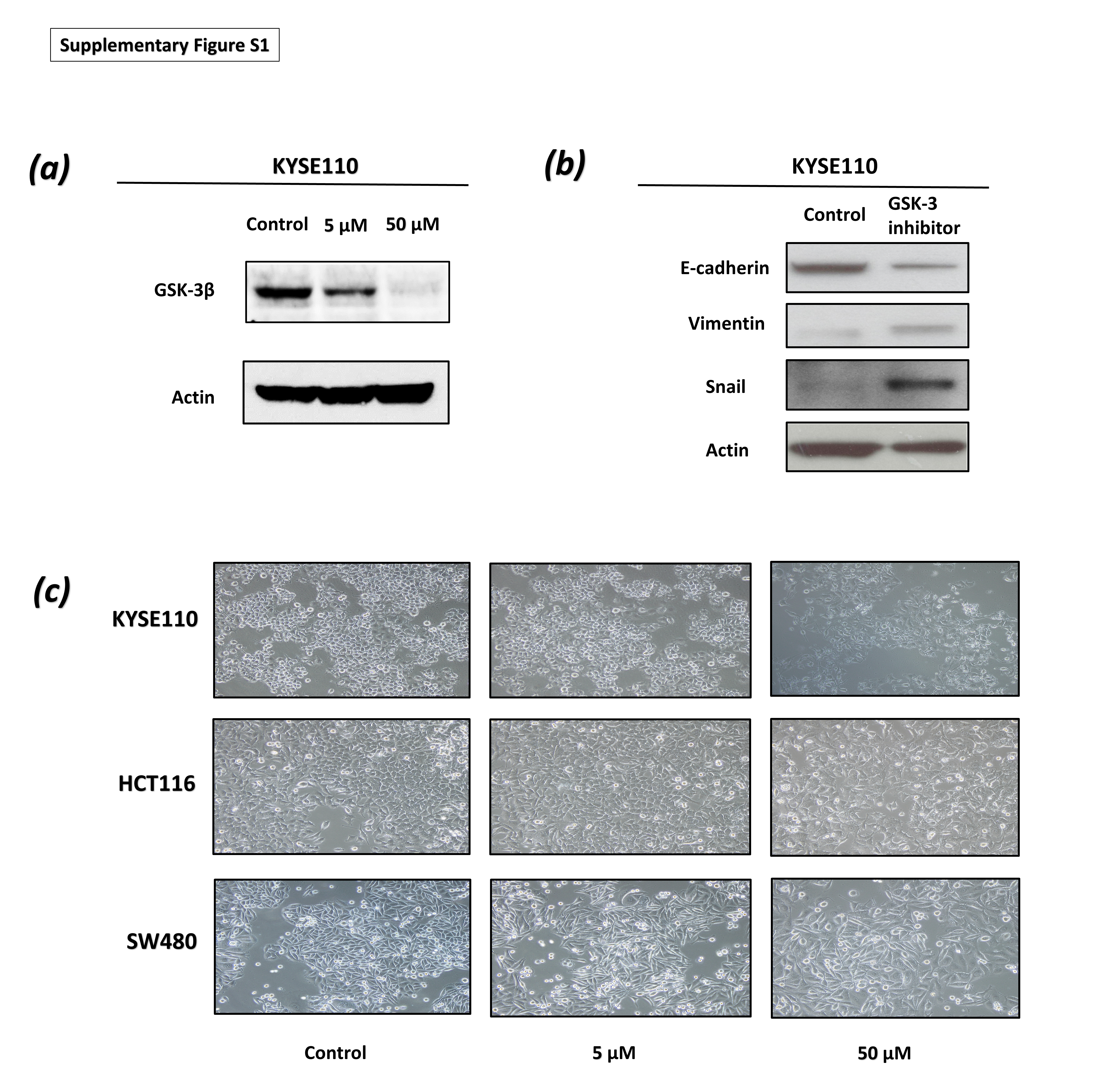

Supplement: Supplementary file 1 [file CAM4-7-3321-s001.tif]

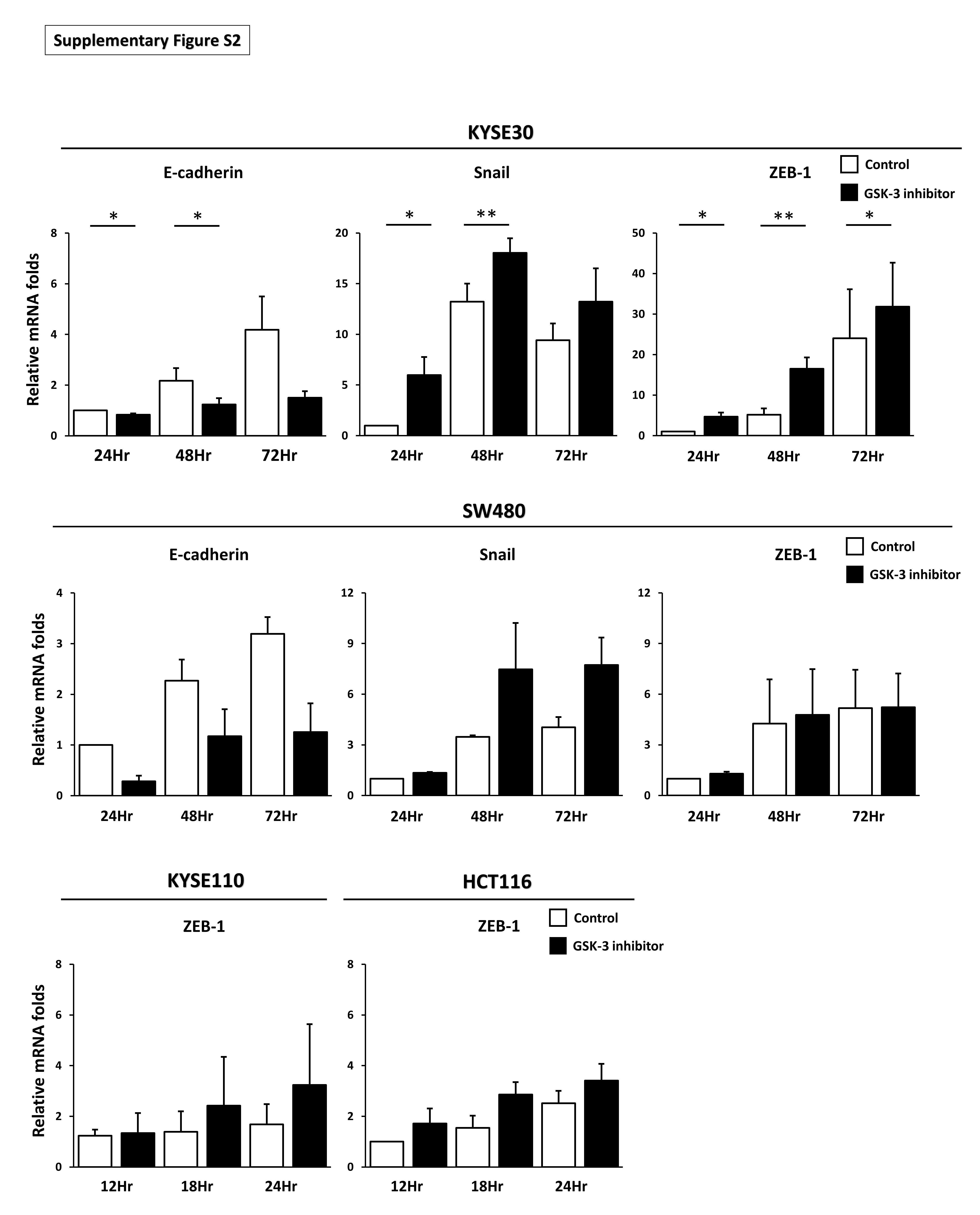

Supplement: Supplementary file 2 [file CAM4-7-3321-s002.tif]

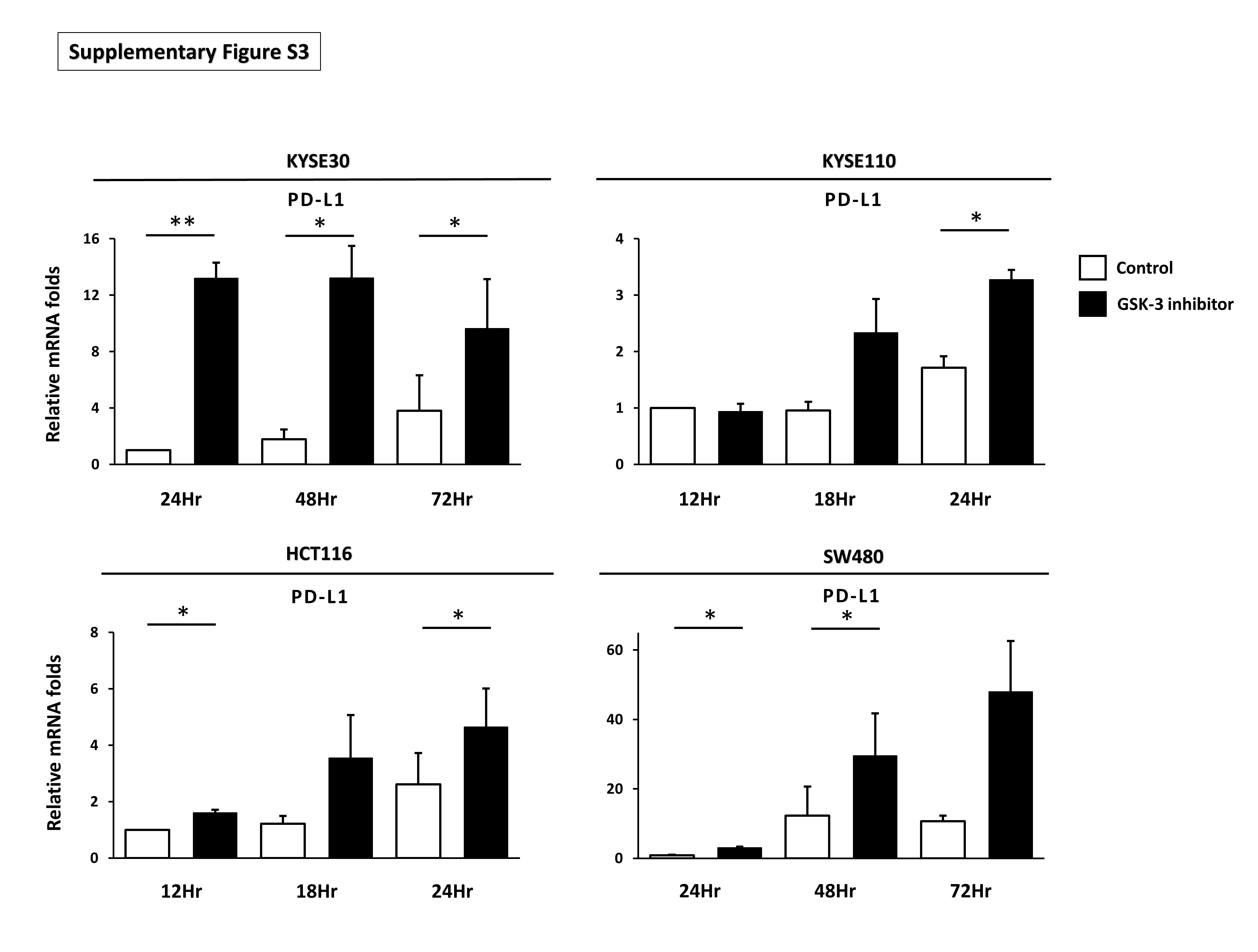

Supplement: Supplementary file 3 [file CAM4-7-3321-s003.tif]

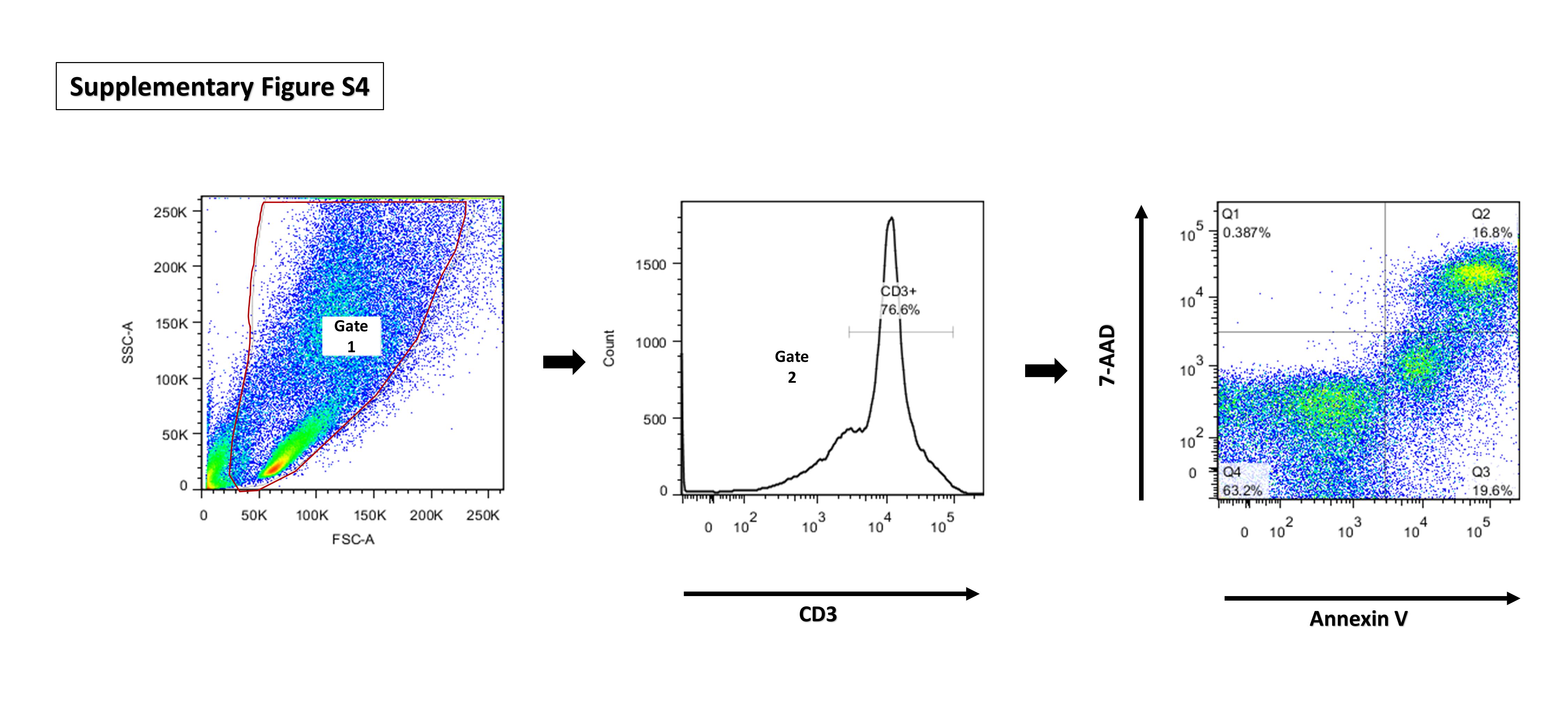

Supplement: Supplementary file 4 [file CAM4-7-3321-s004.tif]

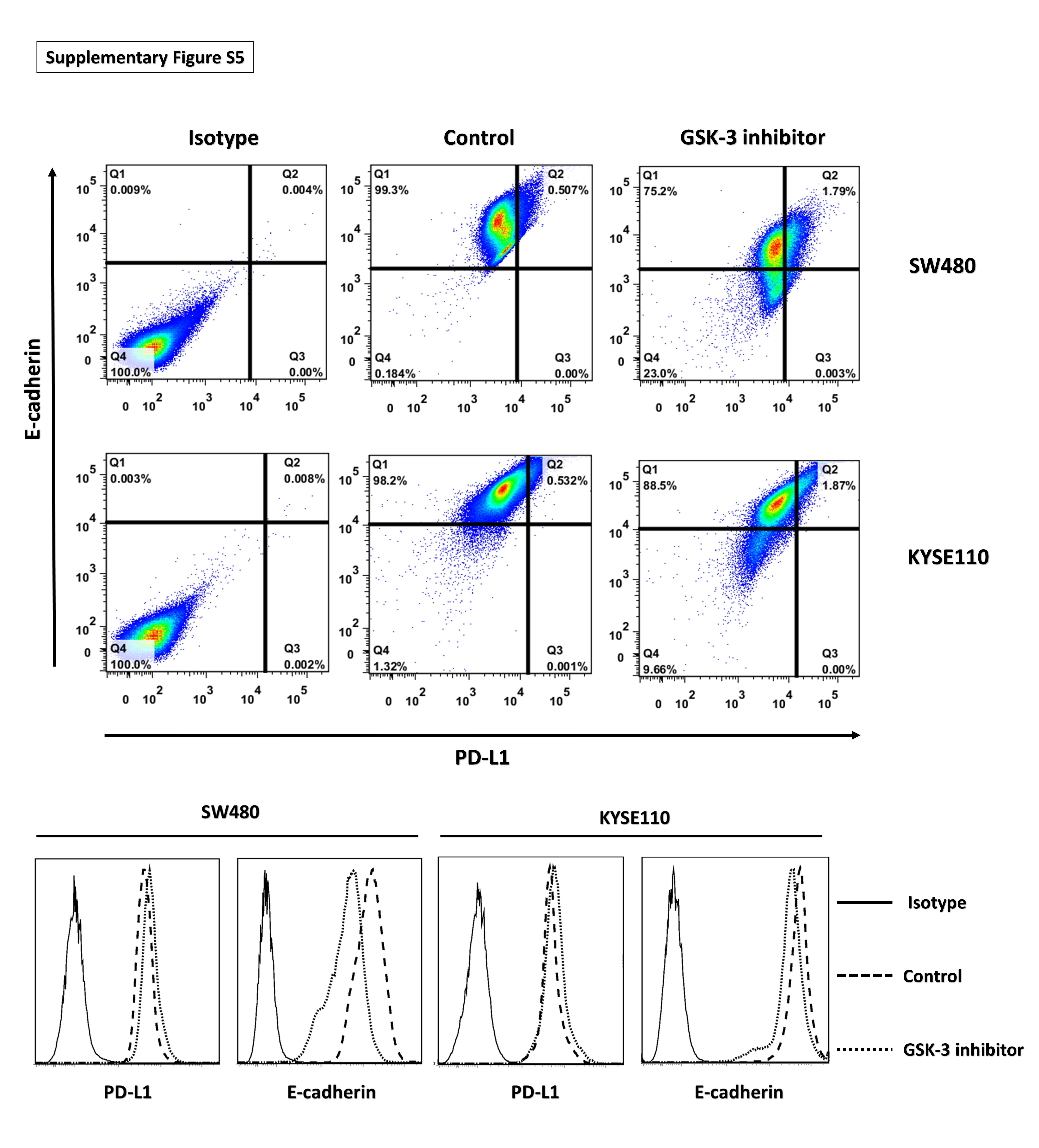

Supplement: Supplementary file 5 [file CAM4-7-3321-s005.TIF]
