# Supplementary material for: Epithelial‐mesenchymal transition‐converted tumor cells can induce T‐cell apoptosis through upregulation of programmed death ligand 1 expression in esophageal squamous cell carcinoma
Source: Cancer Med. 2018 May 31;7(7):3321–30. doi: 10.1002/cam4.1564 (PMC6051241; doi:10.1002/cam4.1564)
Supplement: Supplementary file 6 [file CAM4-7-3321-s006.docx]

**Supporting Figure Legends**
**Supporting Figure S1.** Treatment with GSK-3 inhibitor induces EMT in tumor cell lines.
**(a)** GSK-3 inhibitor, SB-415286, reduces the expression of the GSK-3β protein in a dose-dependent manner in KYSE110 (Control, 5 µM, 50 µM). **(b)** Western blot analysis of KYSE110 showed the EMT status, after exposure to 50 µM of GSK-3 inhibitor for 48 hours. **(c)** Microscopic images showed the morphological changes of KYSE110, HCT116, and SW480 treated with the GSK-3 inhibitor (Control, 5 µM, 50 µM) for 48 hours. In all treated cell lines, spindle-shaped cells with loss of cell to cell adhesion were seen in 50 µM treatment.

**Supporting Figure S2.** Treatment with GSK-3 inhibitor modifies the EMT related genes

Data of QT-PCR analysis on tumor cell lines at different time points after treatment with the 50 µM of GSK-3 inhibitor were shown. The E-cadherin gene expression was down-regulated, while Snail and ZEB-1 gene expression was up-regulated at different time points. DMSO was used for untreated control. ** < 0.05*, ** *< 0.01*.

**Supporting Figure S3.** The up-regulation of PD-L1 in different time points of all cell lines

Data of the PD-L1 QT-PCR analysis on tumor cell lines at different time points after treatment with the 50 µM of GSK-3 inhibitor were shown. The PD-L1 gene expression was up-regulated at different time points. DMSO was used for untreated control. ** < 0.05*, ** *< 0.01*.
 **Supporting Figure S4.** Gating methods of flow cytometry to analyze apoptosis

A left figure showed the first gate including IL-2 activated T cells and tumor cells, where the cells in low FSC and SSC value were excluded. A middle figure showed the histogram under the first gate and the second gate was added to the CD3 positive cells. A right figure was showed under both first and second gate, and we made the quadrant gate where Q1 = dead cells, Q2 = late apoptotic cells, Q3 = early apoptotic cells, and Q4 = viable cells. The X-axis is Annexin V, and Y-axis is 7-AAD. The proportion of apoptotic cells was determined using Annexin V and 7-AAD staining.

**Supporting Figure S5.** The relationship between EMT conversion and PD-L1 expression
The figures showed the downregulation of E-cadherin and the upregulation of PD-L1 on the GSK-3 inhibitor treated samples of SW480 and KYSE110 cell lines, compared to the control samples. DMSO was used for untreated control.
